# Evaluating the sensitivity of the Wako β-D-glucan assay for the diagnosis of candidemia caused by *Candida parapsilosis*

**DOI:** 10.1007/s10096-026-05446-z

**Published:** 2026-03-04

**Authors:** Yuta Kuhara, Hiroki Kitagawa, Kayoko Tadera, Keitaro Omori, Norifumi Shigemoto, Tomoyuki Akita, Shingo Fukuma, Shinya Takahashi, Hiroki Ohge

**Affiliations:** 1 Department of Infectious Diseases, Hiroshima University Hospital, 1-2-3 Kasumi, Minami-ku, Hiroshima, 734- 8551 Japan; 2 Department of Surgery, Graduate School of Biomedical and Health Sciences, Hiroshima University, Hiroshima, 734-8551 Japan; 3 Section of Clinical Laboratory, Division of Clinical Support, Hiroshima University Hospital, Hiroshima, 734-8551 Japan; 4 Division of Laboratory Medicine, Hiroshima University Hospital, Hiroshima, 734-8551 Japan; 5 Translational Research Center, Hiroshima University, Hiroshima, 734-8551 Japan; 6 Department of Epidemiology, Disease Control and Prevention, Graduate School of Biomedical and Health Sciences, Hiroshima University, Hiroshima, 734-8551 Japan

**Keywords:** Candidemia, Candida parapsilosis, Candida albicans, (1,3)-β-D-glucan, Sensitivity and Specificity, Catheter-related bloodstream infection

## Abstract

**Purpose:**

Candidemia poses diagnostic challenges because of non-specific symptoms and low sensitivity of blood cultures. The Wako (1,3)-β-D-glucan (BDG) assay was recently introduced in Europe; however, its diagnostic performance for *Candida parapsilosis* compared to that of the Fungitell assay, which has demonstrated lower sensitivity, remains unclear. We evaluated the diagnostic performance of Wako BDG and BDG levels in candidemia, focusing on *C. parapsilosis*.

**Methods:**

BDG samples obtained within ± 3 days of blood culture collection were retrospectively analyzed. Diagnostic performance was compared using multiple cutoffs, including manufacturer-recommended thresholds (Japan: 11 pg/mL, Europe: 7.0 pg/mL). BDG levels and clinical characteristics were compared between *C. parapsilosis* and non-*parapsilosis Candida* candidemia.

**Results:**

We included 154 candidemia episodes (*C. parapsilosis*: *n* = 24, non-*parapsilosis Candida*: *n* = 130) and 3,856 control episodes. Using 11 pg/mL, sensitivity for *C. parapsilosis* was 38% (95% confidence interval [CI]: 21–57) versus 62% (95% CI: 54–70) for non-*parapsilosis Candida* (*p* = 0.041), with specificity 93%. Lowering the cutoff from 11 to 7.0 pg/mL increased the overall sensitivity from 58% (95% CI: 51–66) to 71% (95% CI: 63–77) while maintaining high specificity (90%) but did not eliminate the sensitivity gap for *C. parapsilosis*. The median BDG level was lower in *C. parapsilosis* (5.0 pg/mL, interquartile range [IQR]: 0–68) than in non-*parapsilosis Candida* (21 pg/mL, IQR: 7.2–97; *p* = 0.036). *C. parapsilosis* candidemia was most commonly catheter-related (67%), with 17% in-hospital mortality.

**Conclusions:**

Despite high specificity, Wako BDG assay showed significantly lower sensitivity and BDG levels for *C. parapsilosis* candidemia compared with non-*parapsilosis Candida* candidemia.

**Supplementary Information:**

The online version contains supplementary material available at 10.1007/s10096-026-05446-z.

## Introduction

Candidemia is a life-threatening infection without specific clinical symptoms. Delayed treatment because of the low sensitivity of blood cultures (~ 50%) and prolonged incubation times contribute to the infection’s high mortality rates [1]. (1,3)-β-D-glucan (BDG) assay provides quick results for identifying invasive fungal infections and is currently recommended by international guidelines for diagnosing invasive candidiasis [2]. Therefore, thoroughly understanding this assay’s efficacy for identifying candidemia is crucial.

Although *Candida albicans* remains the most common causative species of candidemia, its dominance has declined over the past two decades, as the incidence of non-*albicans Candida* infections has increased [3–5]. Among these, *C. parapsilosis* is the second or third most frequently identified causative species. *C. parapsilosis* is clinically significant because of its ability to form biofilms on central venous catheters and other medical devices, thus posing significant risks to patients who undergo invasive procedures [6]. Studies using the Fungitell assay (FA; Associates of Cape Cod, East Falmouth, MA, USA) have reported a lower sensitivity for BDG in detecting *C. parapsilosis* infections vs. infections caused by other *Candida* species [7–9]. The diagnostic performance of BDG has traditionally been evaluated using the FA. In Japan, the Wako BDG test (FUJIFILM Wako Pure Chemical Corporation, Osaka, Japan) has been more widely used. The European Conformity-marked version of the Wako test was recently introduced in Europe. Reports of BDG evaluations in Europe, performed using the Wako assay, have therefore increased [10–12]. However, to our knowledge, there have been no studies published thus far investigating the lower sensitivity of the Wako BDG assay for detecting *C. parapsilosis* infections. Wako and FA assays differ in BDG calibrators (pachyman vs. lentinan), detection principles (turbidimetric vs. colorimetric), and assay-specific interpretive cutoffs (e.g., 7.0 or 11 pg/mL for Wako vs. 80 pg/mL for FA) [13]. Comparative studies using Wako and FA on the same clinical samples have demonstrated assay-dependent differences in diagnostic performances [12–14]. Therefore, direct comparison of absolute BDG values across assays is limited. This study aimed to evaluate the sensitivity of the Wako BDG assay and BDG levels in episodes of candidemia caused by various *Candida* species—with a particular focus on *C. parapsilosis*.

## Materials and methods

### Study population

This retrospective study analyzed episodes where blood culture (BC) was done, identified between January 2008 and December 2024 at Hiroshima University Hospital, Japan. The inclusion criteria for patient enrollment were: a) ≥ 18 years of age; b) episodes in which BCs were performed; and c) episodes with BDG samples that were collected within ± 3 days of the corresponding BCs. We included only BC-proven candidemia episodes based on the European Organization for Research and Treatment of Cancer/Invasive Fungal Infections Cooperative Group and the National Institute of Allergy and Infectious Diseases Mycoses Study Group (EORTC/MSG) criteria [2]. The exclusion criteria were: (a) candidemia caused by ≥ 2 species; (b) invasive fungal infections caused by non-*Candida* fungi; (c) co-infections with *Candida* and other fungal pathogens; and (d) candidemia episodes diagnosed within 2 months from the date of the first negative BC.

### Definitions

The day of BC collection was defined as day 0, and BDG samples taken within ± 3 days of this date were included. In episodes where multiple BDG samples were collected within this window, the sample closest to the corresponding BC was analyzed. The EQUAL Candida score for each episode was determined according to previously published criteria [15]. Nosocomial infections were defined as those that developed more than 48 h after hospital admission.

### Data collection

The data were collected by reviewing medical records. The data collected included demographic patient information (i.e., age and sex), underlying conditions, BC collection details, candidemia etiologies, *Candida* species isolated from BC, and in-hospital mortality.

### Microbiological analysis

All BCs were performed using the BacT/ALERT 3D kit (bioMérieux, Marcy l’Étoile, France) between January 1, 2008 and April 17, 2022; and the BacT/ALERT Virtuo kit (bioMérieux) between April 18, 2022 and December 31, 2024. BacT/ALERT FA and FN Plus bottles (bioMérieux) were used during both periods. The BCs were incubated for a maximum of 7 days. *Candida* species were identified using the Vitek 2 compact system with a YST ID card (bioMérieux) between January 1, 2008 and March 31, 2021, and via matrix-assisted laser desorption/ionization time-of-flight mass spectrometry (MALDI-TOF MS) with a MALDI Biotyper Sirius system (Bruker Daltonik GmbH, Bremen, Germany) between April 1, 2021 and December 31, 2024. All *Candida* species isolates from BCs were stored as glycerol stocks at − 80 °C until they were used. Any *Candida* species identified using the Vitek 2 compact system were re-identified using MALDI-TOF MS.

### BDG evaluation

BDG levels in serum and plasma samples were measured using the Wako assay (FUJIFILM Wako Pure Chemical Corporation). Measurements were taken on an MT-358 Toxinometer (FUJIFILM Wako Pure Chemical Corporation) between January 1, 2008 to February 28, 2012; an MT-5500 Toxinometer (FUJIFILM Wako Pure Chemical Corporation) between March 1, 2012 to June 30, 2024; and an MT-7500 LIMUSAVE (FUJIFILM Wako Pure Chemical Corporation) between July 1, 2024 to December 31, 2024; in accordance with the manufacturer’s instructions. The correlation of BDG measurements across this series of devices has been validated [16]. BDG values ≥ 11 pg/mL were considered positive, as indicated by the manufacturer’s instructions for the Japanese devices. In 2021, a newly recommended cutoff of ≥ 7.0 pg/mL was introduced in Europe [11]. We therefore evaluated this cutoff alongside the original one. Two different measurement modes were used during the study period: quantitative (January 1, 2008 – October 31, 2016), and threshold (November 1, 2016 – December 31, 2024). In the threshold mode, results limit the quantifiable range to 6–600 pg/mL and 1–600 pg/mL for the MT-358 and MT-5500 Toxinometer and MT-7500 LMUSAVE, respectively. In the threshold mode, values below the limit of quantification were considered 0 pg/mL for analysis following a previous method [13]. Samples with BDG concentrations > 600 pg/mL were serially diluted and remeasured. In the quantitative mode, assay-specific calibration curves were used to define the lower limit of quantification (LOQ), allowing numeric values to be obtained even for samples outside the threshold-mode range. Measurable values were analyzed, when available, and values below the assay-specific LOQ were considered 0 pg/mL.

### Statistical analysis

Statistical analyses were performed using JMP Pro version 18.0.1 (SAS Institute Inc., Cary, NC). Because multiple candidemia episodes from the same individuals were included, inferential statistical comparisons were not performed, and the results are presented descriptively. Sensitivity, specificity, positive predictive value, and negative predictive value at each BDG cutoff were calculated from 2 × 2 contingency tables using BC-confirmed candidemia as the reference standard. Receiver operating characteristic (ROC) curves were plotted for the Wako BDG assay to assess diagnostic performance across cutoffs, and the optimal cutoff was determined by maximizing the Youden index. All 95% confidence intervals (CI) were calculated using the Wilson scoring method. Statistical significance was defined as *p* < 0.05.

## Results

**Fig. 1:**
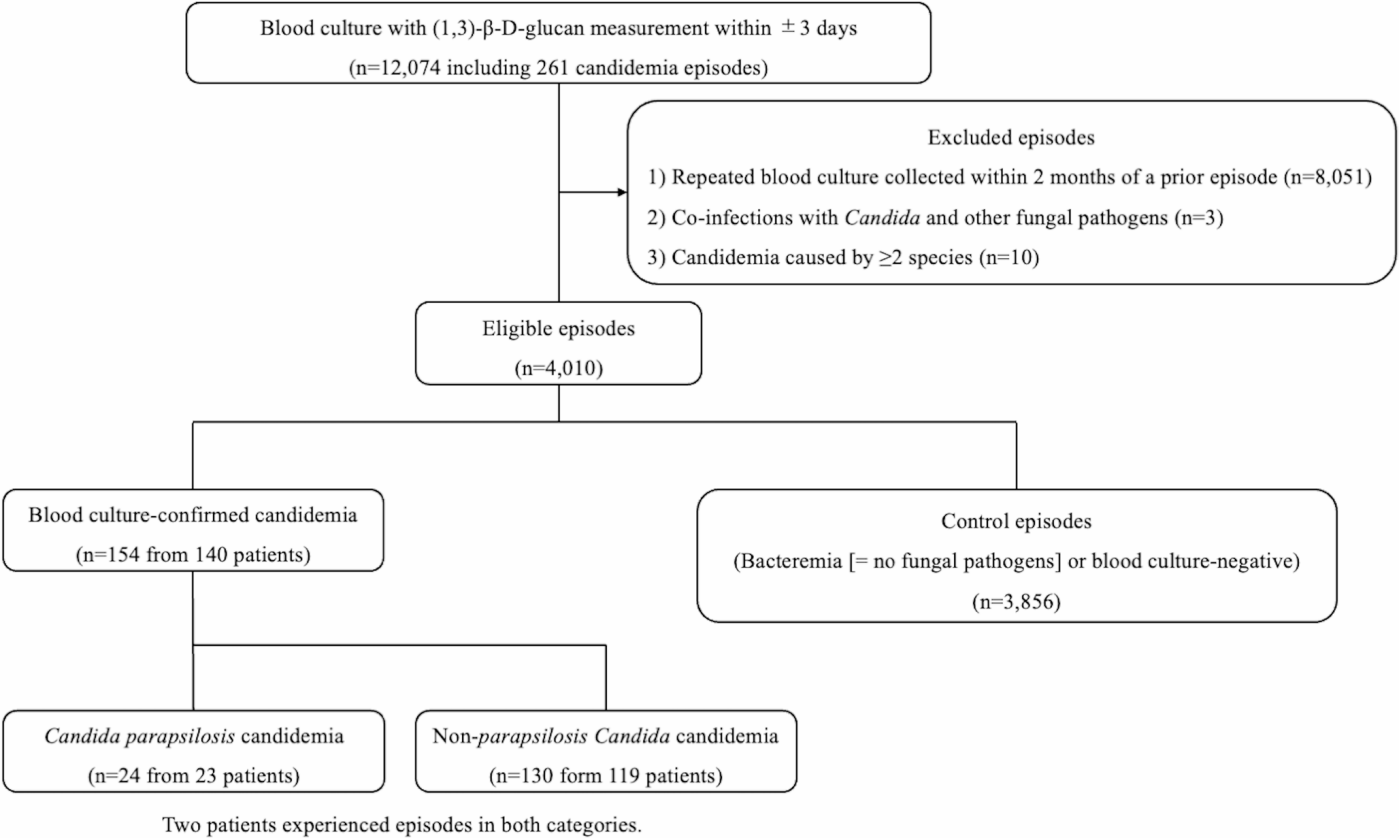
Flow diagram. Two patients experienced episodes in both candidemia groups

A total of 12,074 BC sampling episodes from adult patients with BDG measured within ± 3 days were identified, of which 4,010 episodes met the inclusion criteria (Fig. 1). Among these, 154 episodes from 140 unique patients were classified as candidemia (*C. parapsilosis*: *n* = 24, non-*parapsilosis Candida*: *n* = 130; Online Resource 1). The remaining 3,856 episodes were non-candidemia controls (bacteremia or BC-negative). Nine patients experienced multiple episodes of candidemia: two patients had six and three episodes, respectively, and the remaining seven patients had two episodes each. All episodes were classified as new because appropriate source control and adequate antifungal therapy were implemented for each of them, and more than 2 months had elapsed since BC clearance, as confirmed by two infectious disease specialists (HK and KO). Of these episodes, eight were caused by different *Candida* species and 15 by the same species (Online Resource 2).

The Wako BDG assay performance varied according to the cutoff used; the corresponding 2 × 2 contingency tables using BC-confirmed candidemia as the reference standard are presented in Table 1. At the 11 pg/mL cutoff, the sensitivity for *C. parapsilosis*, non-*parapsilosis Candida* and *C. albicans* was 38% (95% CI: 21–57), 62% (95% CI: 54–70), and 66% (95% CI: 55–76), respectively, with the corresponding specificity of 93% (95% CI: 92–94). Lowering the BDG cutoff from 11 to 7.0 pg/mL increased overall sensitivity from 58% to 71% while maintaining high specificity (93% at 11 pg/mL and 90% at 7.0 pg/mL; Table 1). This increase was primarily attributed to higher detection of non-*parapsilosis Candida*.

**Table 1:** Sensitivity and specificity of (1,3)-β-D-glucan within ± 3 days from collected blood cultures at various cutoff values

All Candida species (154 candidemia episodes; 3856 control episodes)								
BDG cutoff (pg/mL)	TP	FN	FP	TN	Sensitivity (95% CI)	Specificity (95% CI)	PPV	NPV
≥ 7	110	44	386	3470	71% (64–78)	90% (89–91)	22%	98.7%
≥ 9	97	57	307	3549	63% (55–70)	92% (91–93)	24%	98.4%
≥ 11	90	64	258	3598	58% (52–66)	93% (92–94)	26%	98.3%
≥ 13	85	69	226	3630	55% (47–63)	94% (93–95)	27%	98.1%
*Candida parapsilosis* (24 candidemia episodes; 3856 control episodes)								
BDG cutoff (pg/mL)	TP	FN	FP	TN	Sensitivity (95% CI)	Specificity (95% CI)	PPV	NPV
≥ 7	10	14	386	3470	42% (25–61)	90% (89–91)	3%	99.6%
≥ 9	9	15	307	3549	38% (21–57)	92% (91–93)	3%	99.6%
≥ 11	9	15	258	3598	38% (21–57)	93% (92–94)	3%	99.6%
≥ 13	9	15	226	3630	38% (21–57)	94% (93–95)	4%	99.6%
Non-*parapsilosis Candida* (130 candidemia episodes; 3856 control episodes)								
BDG cutoff (pg/mL)	TP	FN	FP	TN	Sensitivity (95% CI)	Specificity (95% CI)	PPV	NPV
≥ 7	100	30	386	3470	77% (69–83)	90% (89–91)	21%	99.1%
≥ 9	88	42	307	3549	68% (59–75)	92% (91–93)	22%	98.8%
≥ 11	81	49	258	3598	62% (54–70)	93% (92–94)	24%	98.7%
≥ 13	76	54	226	3630	58% (50–67)	94% (93–95)	25%	98.5%
*Candida albicans* (77 candidemia episodes; 3856 control episodes)								
BDG cutoff (pg/mL)	TP	FN	FP	TN	Sensitivity (95% CI)	Specificity (95% CI)	PPV	NPV
≥ 7	61	16	386	3470	79% (69–87)	90% (89–91)	14%	99.5%
≥ 9	55	22	307	3549	71% (61–80)	92% (91–93)	15%	99.4%
≥ 11	51	26	258	3598	66% (55–76)	93% (92–94)	17%	99.3%
≥ 13	45	32	226	3630	58% (47–69)	94% (93–95)	17%	99.1%

Blood culture-confirmed candidemia (positive) vs. control episodes (negative; bacteremia or blood culture-negative). TP/FN were derived from the number of BDG-positive episodes among blood culture-confirmed candidemia at each cutoff; FP/TN were derived from BDG results in 3856 control episodes

BDG, (1,3)-β-D-glucan

*TP* true positive, *FN* false negative, *FP* false positive, *TN* true negative, *CI* confidence interval, *PPV* positive predictive value, *NPV* negative predictive value

**Fig. 2:**
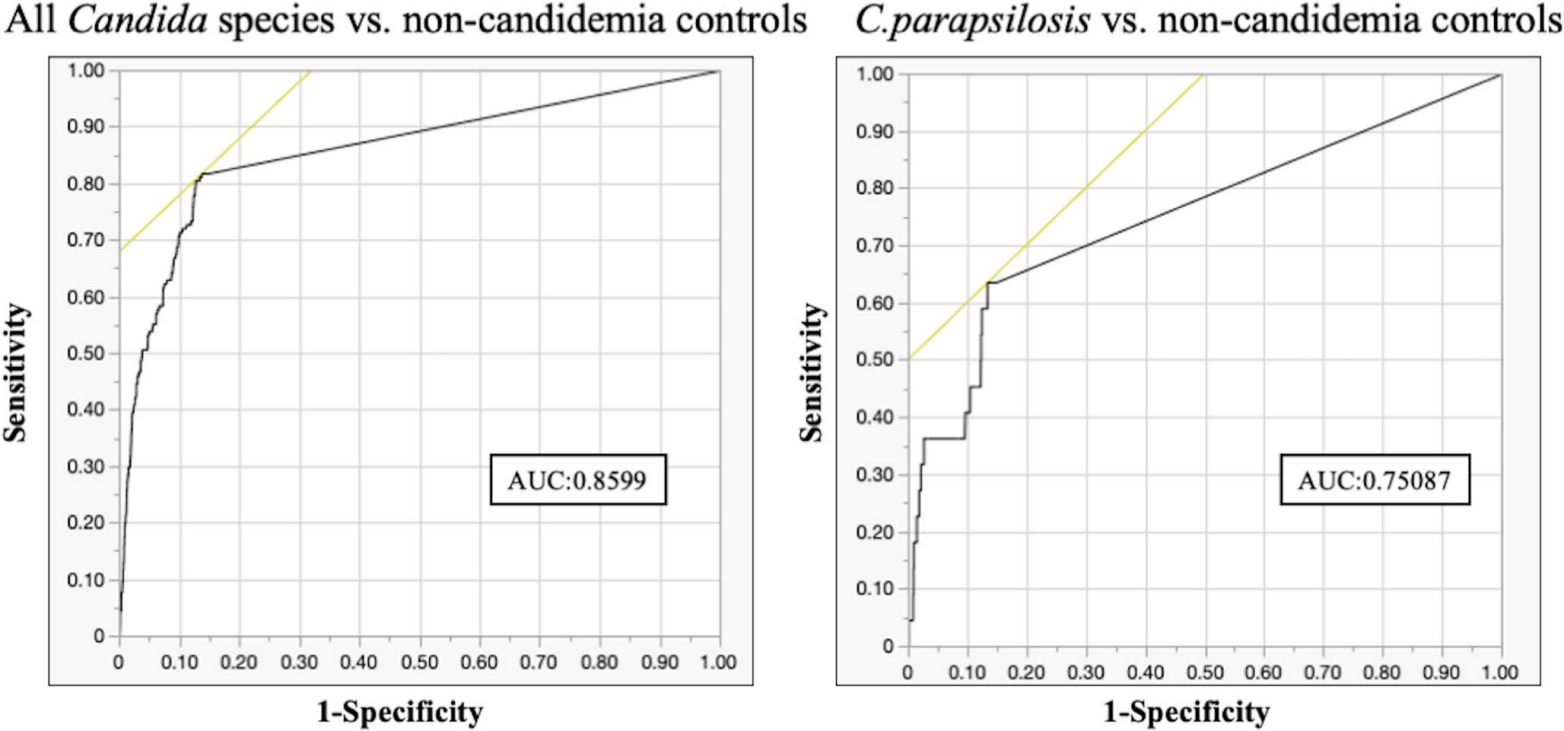
Receiver operating characteristic (ROC) curves of the Wako (1,3)-β-D-glucan assay. Left panel, discrimination of all *Candida* candidemia vs. non-candidemia controls (Area under curve: AUC 0.860); right panel, discrimination of *C. parapsilosis* candidemia vs. non-candidemia controls (AUC 0.751). The y-axis shows sensitivity, and the x-axis shows 1–specificity; The intersection of each ROC curve with the yellow guideline marks the cutoff that maximizes the Youden index

ROC curve analysis was performed to evaluate the potential for improved diagnostic accuracy (Fig. 2). The highest Youden index for all *Candida* episodes was 0.86 at a cutoff of 2.4 pg/mL (sensitivity 82%, specificity 86%). For *C. parapsilosis* episodes, the highest Youden index was 0.75 at a cutoff of 2.7 pg/mL (sensitivity 63%, specificity 87%).

**Fig. 3:**
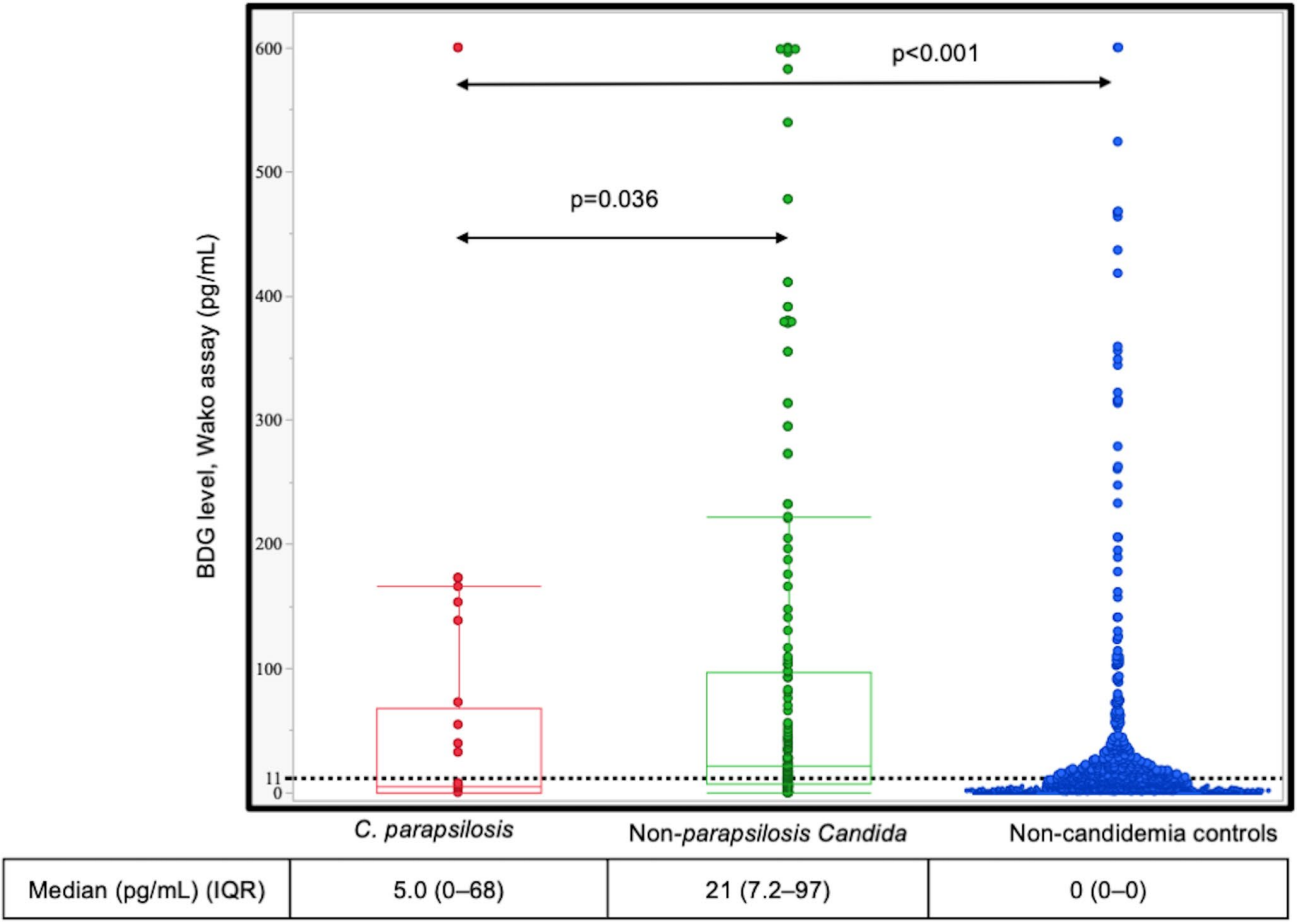
(1,3)-β-D-glucan (BDG) concentrations (pg/mL) for *C. parapsilosis* candidemia, non-*parapsilosis Candida* candidemia, and non-candidemia controls. Dots represent individual episodes; boxes denote interquartile ranges (IQR) with horizontal lines indicating medians. The horizontal black dotted lines mark the manufacture’s cutoff of 11 pg/mL. Pairwise comparisons yielded the p-values indicated on the figure; medians (IQR) were 5.0 (0–68) pg/mL for *C. parapsilosis*, 21 (7.2–97) pg/mL for non-*parapsilosis Candida*, and 0 (0–0) pg/mL for non-candidemia controls

Box plots of the BDG results are presented in Fig. 3. Numeric values of BDG were obtained for all samples reported as > 600 pg/mL (one, three, and four episodes in *C. parapsilosis*, non-*parapsilosis Candida*, and controls, respectively). Conversely, samples below the LOQ did not yield numeric values, comprising 9 *C. parapsilosis*, 19 non-parapsilosis *Candida*, and 3,280 control episodes. The median BDG concentrations were 5.0 pg/mL (IQR: 0–68 pg/mL) for *C. parapsilosis* candidemia, 21 pg/mL (IQR: 7.2–97 pg/mL) for non-*parapsilosis Candida* candidemia, and 0 pg/mL (IQR: 0–0 pg/mL) for controls (*p* < 0.001).

A subset of 154 episodes with candidemia was further analyzed. Patient- and episode-level characteristics of candidemia caused by *C. parapsilosis*, non-*parapsilosis Candida*, and *C. albicans* are summarized in Table 2. The proportion of BDG-positive episodes was lower in the *C. parapsilosis* group compared with the non-parapsilosis *Candida* group. Catheter-related bloodstream infection (CRBSI) was more frequently observed among episodes of *C. parapsilosis* candidemia. In contrast, episodes caused by *C. parapsilosis* were less frequently associated with intensive care unit (ICU) management, abdominal infection, administration of intravenous immunoglobulin (IVIG) or albumin within 7 days, and in-hospital mortality. The *C. albicans* group showed trends similar to those of non-*parapsilosis Candida*.

**Table 2:** Patient- and episode-level characteristics of candidemia caused by *Candida parapsilosis*, non-*parapsilosis Candida*, and *Candida albicans*, stratified by (1,3)-β-D-glucan levels measured within ± 3 days of blood culture

Patient-level characteristics	C. parapsilosis	Non-parapsilosis Candida	C. albicans
	23 patients	119 patients	71 patients
Age (years), median (IQR)	58 (37–68)	68 (58–75)	67 (52–75)
Male	15 (67%)	77 (65%)	44 (62%)
Underlying disease			
Solid tumor	4 (17%)	33 (28%)	16 (23%)
Hematologic tumor	4 (17%)	13 (11%)	3 (4.2%)
Transplant patients	1 (4.4%)	20 (17%)	9 (13%)
Immunosuppressant drug user	3 (13%)	28 (24%)	16 (23%)
Hemodialysis	3 (13%)	37 (31%)	28 (39%)
Episode-level characteristics (*n* = 154)	24 episodes	130 episodes	77 episodes
Nosocomial infection	20 (83%)	113 (87%)	69 (90%)
BDG (pg/mL), median (IQR)	5.0 (0–68)	21 (7.2–97)	21 (7.8–106)
Serum or plasma sample			
Serum sample	19 (79%)	105 (81%)	62 (81%)
Positive			
>11 pg/mL	9 (38%)	81 (62%)	51 (66%)
> 7.0 pg/mL	10 (42%)	99 (76%)	61 (79%)
<6.0 pg/mL	13 (54%)	28 (22%)	15 (19%)
Context of blood culture sampling			
Intensive Care Unit stay	4 (17%)	68 (52%)	45 (58%)
Central venous catheter	20 (83%)	107 (82%)	64 (83%)
After abdominal surgery hospitalization	3 (13%)	40 (31%)	23 (30%)
Antibiotic therapy within 7 days	14 (58%)	103 (79%)	64 (83%)
Antifungal agent therapy within 7 days	4 (17%)	28 (22%)	17 (22%)
IVIG administration within 7 days	0 (0%)	33 (25%)	21 (27%)
Albumin administration within 7 days	6 (25%)	67 (52%)	46 (60%)
Etiology of candidemia			
CRBSI	16 (67%)	61 (47%)	39 (51%)
Abdominal infection	1 (4.2%)	34 (26%)	20 (26%)
Febrile neutropenia	2 (8.3%)	4 (3.1%)	2 (2.6%)
Chest infection	0 (0%)	4 (3.1%)	2 (2.6%)
Burn wound	0 (0%)	3 (2.3%)	1 (1.3%)
Infectious endocarditis	1 (4.2%)	2 (1.5%)	1 (1.3%)
Others	0 (0%)	10 (7.7%)	7 (9.0%)
Unknown	4 (17%)	12 (9.2%)	5 (6.5%)
Endophthalmitis *Candida*	0 (0%)	12 (9.2%)	9 (12%)
EQUAL Candida Score ≥ 10	18 (75%)	114 (88%)	69 (90%)
In-hospital mortality	4 (17%)	70 (54%)	47 (61%)

To analyze patient characteristics, episodes were considered independent observations within each species group, even when they occurred in the same individual. When multiple episodes from the same patient occurred within the same group, only the first episode was included in the analysis. Two patients experienced candidemia episodes in both the *C. parapsilosis* and non-parapsilosis *Candida* groups

*IQR* interquartile range, *BDG* (1,3)-β-D-glucan, *IVIG* intravenous immunoglobulin, *CRBSI* catheter related blood stream infection

## Discussion

In this study, BDG levels were significantly lower in the episodes of candidemia caused by *C. parapsilosis* than in those caused by other *Candida* species, when the Wako assay was used—highlighting a species-specific difference in BDG detection. This finding is consistent with previous reports on the FA, which demonstrated lower sensitivity for *C. parapsilosis* and *C. auris* vs. other *Candida* species [7, 8, 17]. In our study, the overall distribution of *Candida* species was comparable with that described in a previous Japanese report [4], but no *C. auris* was detected (Online Resource 1). The biological basis for lower BDG detectability in *C. parapsilosis* is unclear. The reduced sensitivity of BDG assays in *C. parapsilosis* candidemia may reflect species-specific differences in cell wall composition, including lower β-1,3-glucan content or reduced release into the circulation compared with other *Candida* species [8]. Moreover, *C. parapsilosis* exhibits intrinsically higher echinocandin minimum inhibitory concentrations, likely related to differences in β-1,3-glucan synthase regulation, which may indirectly affect cell wall β-1,3-glucan architecture and exposure [5, 18, 19]. However, we did not directly assess cell-wall β-1,3-glucan content, BDG shedding, or effects of antifungal exposure.

The sensitivity of the Wako BDG assay for *C. parapsilosis* was 38%, lower than that for non-*parapsilosis Candida* species (62%) and *C. albicans* (66%). While the Wako assay maintained high specificity across cutoffs (approximately 90–94%), its sensitivity was substantially reduced for *C. parapsilosis*; therefore, an early negative BDG result should not be used to rule out *C. parapsilosis* candidemia. Candidemia should not be ruled out solely based on an early negative BDG result because BDG levels may exhibit delayed elevation [19]; consequently, repeat BDG testing is warranted in clinically suspected cases. Prior studies using the FA have also reported reduced BDG sensitivity for *C. parapsilosis* [7]. Conversely, Dichtl et al. reported that the Wako BDG assay exhibited a higher sensitivity for *C. parapsilosis* (73%) than observed in our study (38%) [10]. Although both studies focused exclusively on BC-confirmed episodes defined according to the EORTC/MSG criteria, certain notable methodological differences included the timing of BDG measurements (our ± 3 days vs. their 6 days before), specimen (serum and plasma vs. serum only), and the BC system used. Previous studies demonstrated that the timing of BDG measurement relative to BC collection did not significantly affect sensitivity, and serum and plasma values obtained with the Wako assay are generally comparable [11, 20]. Therefore, differences in BC systems and associated organism recovery characteristics may have contributed to variability in observed diagnostic performance. Our study used the BacT/ALERT system (bioMérieux), whereas Dichtl et al. used the BD BACTEC FX system (Becton, Dickinson and Company). BC systems can differ in yeast recovery and time-to-detection; for instance, in a comparative study, in a yeast-spiking experiment [21], BacT/ALERT detected growth more frequently than BACTEC, particularly for non-*albicans Candida* species, suggesting that BacT/ALERT may capture episodes with lower organism burdens [21]. If so, cohorts identified using BacT/ALERT could include more low-burden candidemia episodes with lower circulating BDG levels, potentially reducing apparent BDG sensitivity [11, 21]. However, because fungal burden was not quantified and the diagnostic performance of the two BC systems for candidemia was not directly compared in this study, this explanation remains hypothesis-generating and should be interpreted with caution.

Consistent with previous reports [6, 22], CRBSI (67%) was the most common cause of *C. parapsilosis* candidemia and *C. parapsilosis* candidemia was associated with lower in-hospital mortality compared with non-*parapsilosis Candida* candidemia. Conversely, abdominal infections caused by *C. parapsilosis* (4.2%) were less frequent than those caused by non-*parapsilosis Candida* (26%). In our cohort, *C. parapsilosis* episodes were also characterized by fewer ICU stays (17%) and less frequent IVIG/albumin administration (0%/25%, respectively) than non-*parapsilosis Candida* candidemia, underscoring heterogeneity in clinical context between species. Because several clinical exposures and healthcare-related factors (including IVIG or albumin administration, surgical gauze exposure, and hemodialysis) increase BDG levels [23], the lower prevalence of these potential BDG-elevating exposures in the *C. parapsilosis* group could partly influence the observed between-species differences in measured BDG distributions. However, because this study was not designed to evaluate the relationship between BDG levels and clinical severity, we could not conclude that these factors were unrelated to the absence of BDG elevation.

The sensitivity of the Wako BDG assay for candidemia was 55–71% in this study, which is consistent with previous findings [10, 11, 13]. To improve its sensitivity, previous studies have suggested lowering the cutoff from 11 to 7.0 pg/mL, which increased the sensitivity from 47% to 59%, and from 67% to 73% [10, 11]. Notably, even after applying the modified cutoff values used in previous studies [10–12], the specificity of the Wako BDG assay remained high (85–93%). Similarly, in our study, applying a cutoff of 7.0 pg/mL improved sensitivity from 58% to 71%, while the specificity remained stable at 90–93%. In addition, ROC analysis yielded an area under the curve of 0.86, with an optimal cutoff of ≥ 2.4 pg/mL within ± 3 days of BC collection (sensitivity 82%, specificity 86%). This lower cutoff elevated the assay’s sensitivity with only a slight decrease in specificity, similarly to a previously reported cutoff of 3.8 pg/mL [13]. Importantly, based on a previous study [13], values below the LOQ were considered 0 pg/mL in this study, and a substantial number of episodes fell into this category. However, the manufacturer-recommended cutoff remains at 11 pg/mL in Japan, as this has been determined to maximize the positive likelihood ratio to 103 [24]. In this study, the positive likelihood ratio was 8.7 at a cutoff of 11 pg/mL, and 7.1 at one of 7.0 pg/mL. Previous studies have reported both increases and decreases in the positive likelihood ratio when the cutoff value was lowered from 11 to 7.0 pg/mL, indicating significant variability in its diagnostic impact [10–12].

A strength of this study is that diagnostic performance (sensitivity and specificity) was evaluated in a comprehensive cohort including all clinical episodes where BCs were obtained and BDG was measured within ± 3 days, rather than a selectively sampled subset. However, this study was subject to several key limitations worth noting. First, this was a retrospective, non-randomized, single-center study with inherent selection bias. Second, we only investigated episodes of candidemia. Candidiasis other than candidemia, as defined by the EORTC/MSG criteria [2], was not included. Third, both serum and plasma samples were included, which may have introduced variability in BDG measurements due to mixing of specimen types. Although plasma is the recommended specimen for the Wako BDG assay, comparable BDG values between the serum and plasma have been obtained [20]. Finally, BDG results below the assay’s lower LOQ were considered 0 pg/mL, consistent with a previous study [13]. This approach may have influenced the distribution of low BDG levels and thus analyses based on quantitative BDG levels, including ROC curves and optimal cutoff values. However, it is unlikely to affect sensitivity and specificity calculated at prespecified dichotomous cutoffs (e.g., 7.0/11 pg/mL), because these metrics are based on binary classification.

In conclusion, the Wako BDG assay is characterized by a significantly lower sensitivity to *C. parapsilosis* candidemia vs. non-*parapsilosis Candida* and *C. albicans* candidemia, despite maintaining high specificity. Thus, BDG should be interpreted as an adjunctive test, and an early negative result should not be used to rule out *C. parapsilosis* candidemia; repeat testing may be considered when clinical suspicion persists. Optimizing the cutoff levels for BDG (i.e., to 7.0 pg/mL) may improve the assay’s sensitivity for detecting candidemia, without compromising its specificity.

## Supplementary Information

Supplementary Material 1 (DOCX 18.9 KB)

## Data Availability

The datasets used or analyzed in the current study are available from the corresponding author upon reasonable request.

## References

[1] Clancy CJ, Nguyen MH (2018) Diagnosing invasive candidiasis. J Clin Microbiol 56:e01909–e01917. 10.1128/jcm.01909-17 29444828 10.1128/JCM.01909-17 PMC5925725

[2] Donnelly JP, Chen SC, Kauffman CA, Steinbach WJ, Baddley JW, Verweij PE et al (2020) Revision and update of the consensus definitions of invasive fungal disease from the European Organization for Research and Treatment of Cancer and the mycoses study group education and research consortium. Clin Infect Dis 71:1367–1376. 10.1093/cid/ciz1008 31802125 10.1093/cid/ciz1008 PMC7486838

[3] Koehler P, Stecher M, Cornely OA, Koehler D, Vehreschild MJGT, Bohlius J et al (2019) Morbidity and mortality of candidaemia in Europe: an epidemiologic meta-analysis. Clin Microbiol Infect 25:1200–1212. 10.1016/j.cmi.2019.04.024 31039444 10.1016/j.cmi.2019.04.024

[4] Kajihara T, Yahara K, Nagi M, Kitamura N, Hirabayashi A, Hosaka Y et al (2022) Distribution, trends, and antifungal susceptibility of Candida species causing candidemia in Japan, 2010–2019: A retrospective observational study based on national surveillance data. Med Mycol 60:myac071. 10.1093/mmy/myac071 36095139 10.1093/mmy/myac071 PMC9521341

[5] Beyer R, Spettel K, Zeller I, Lass-Flörl C, Achleitner D, Krause R et al (2019) Antifungal susceptibility of yeast bloodstream isolates collected during a 10-year period in Austria. Mycoses 62:357–367. 10.1111/myc.12892 30636016 10.1111/myc.12892

[6] Tóth R, Nosek J, Mora-Montes HM, Gabaldon T, Bliss JM, Nosanchuk JD et al (2019) Candida parapsilosis: from genes to the bedside. Clin Microbiol Rev 32:e00111–e00118. 10.1128/cmr.00111-18 30814115 10.1128/CMR.00111-18 PMC6431126

[7] Mikulska M, Giacobbe DR, Furfaro E, Mesini A, Marchese A, Del Bono V et al (2016) Lower sensitivity of serum (1,3)-β-d-glucan for the diagnosis of candidaemia due to Candida parapsilosis. Clin Microbiol Infect 22:646e5–646e8. 10.1016/j.cmi.2016.05.020 10.1016/j.cmi.2016.05.020 27256062

[8] Ostrosky-Zeichner L, Alexander BD, Kett DH, Vazquez J, Pappas PG, Saeki F et al (2005) Multicenter clinical evaluation of the (1–>3) beta-D-glucan assay as an aid to diagnosis of fungal infections in humans. Clin Infect Dis 41:654–659. 10.1086/432470 16080087 10.1086/432470

[9] Del Bono V, Delfino E, Furfaro E, Mikulska M, Nicco E, Bruzzi P et al (2011) Clinical performance of the (1,3)-β-D-glucan assay in early diagnosis of nosocomial Candida bloodstream infections. Clin Vaccine Immunol 18:2113–2117. 10.1128/CVI.05408-11 21994353 10.1128/CVI.05408-11 PMC3232688

[10] Dichtl K, Seybold U, Wagener J (2019) Serological biomarkers of candidemia: a retrospective evaluation of three assays. Infection 47:217–224. 10.1007/s15010-018-1224-3 30264200 10.1007/s15010-018-1224-3

[11] Forster J, Dichtl K, Wagener J (2022) Lower beta-1,3-D-glucan testing cut-offs increase sensitivity for non-albicans Candida species bloodstream infections. Mycoses 65:500–507. 10.1111/myc.13421 35020235 10.1111/myc.13421

[12] De Carolis E, Marchionni F, Torelli R, Angela MG, Pagano L, Murri R et al (2020) Comparative performance evaluation of Wako β-glucan test and Fungitell assay for the diagnosis of invasive fungal diseases. PLoS ONE 15:e0236095. 10.1371/journal.pone.0236095 32726358 10.1371/journal.pone.0236095 PMC7390339

[13] Friedrich R, Rappold E, Bogdan C, Held J (2018) kako J Clin Microbiol 56:e00464–e00418. 10.1128/jcm.00464-18 10.1128/JCM.00464-18 PMC6113455 29899003

[14] Singh S, Kanaujia R, Agnihotri S, Kaur H, Chakrabarti A, Rudramurthy SM (2022) The comparative evaluation of the Fujifilm Wako β-glucan assay and Fungitell assay for diagnosing invasive fungal disease. J Fungi (Basel) 9:6. 10.3390/jof9010006 36675827 10.3390/jof9010006 PMC9861801

[15] Mellinghoff SC, Hoenigl M, Koehler P, Kumar A, Lagrou K, Lass-Flörl C et al (2018) EQUAL Candida Score: an ECMM score derived from current guidelines to measure QUAlity of Clinical candidaemia Management. Mycoses 61:326–330. 10.1111/myc.12746 29325218 10.1111/myc.12746

[16] Tsutomu F, Naoko M, Kyoichi S (2022) Basic study of a (1→3)-β-D-glucan assay using colorimetric method on LIMUSAVE MT-7500. J Anal Bio-sci (Seibutsu Shiryo Bunseki) 5:199–204. https://j-jabs.umin.jp/45/45.199.pdf

[17] Mikulska M, Ullah N, Magnasco L, Codda G, Bartalucci C, Miletich F et al (2024) Lower (1,3)-beta-d-glucan sensitivity and in vitro levels in Candida auris and Candida parapsilosis strains. Clin Microbiol Infect 30:822–827. 10.1016/j.cmi.2024.02.012 38431255 10.1016/j.cmi.2024.02.012

[18] Ostrosky-Zeichner L, Rex JH, Pappas PG, Hamill RJ, Larsen RA, Horowitz HW et al (2003) Antifungal susceptibility survey of 2,000 bloodstream Candida isolates in the United States. Antimicrob Agents Chemother 47:3149–3154. 10.1128/aac.47.10.3149-3154.2003 14506023 10.1128/AAC.47.10.3149-3154.2003 PMC201160

[19] Pappas PG, Kauffman CA, Andes DR, Clancy CJ, Marr KA, Ostrosky-Zeichner L et al (2016) Clinical Practice Guideline for the Management of Candidiasis: 2016 Update by the Infectious Diseases Society of America. Clin Infect Dis 15:62. 10.1093/cid/civ933 10.1093/cid/civ933 PMC4725385 26679628

[20] De Carolis E, Marchionni F, Torelli R, Posteraro P, De Pascale G, Carelli S et al (2019) Comparable serum and plasma 1,3-β-d-Glucan values obtained using the Wako β-glucan test in patients with probable or proven fungal diseases. J Clin Microbiol 57:e00149–e00119. 10.1128/jcm.00149-19 30842228 10.1128/JCM.00149-19 PMC6498008

[21] Horvath LL, George BJ, Murray CK, Harrison LS, Hospenthal DR (2004) Direct comparison of the BACTEC 9240 and BacT/ALERT 3D automated blood culture systems for candida growth detection. J Clin Microbiol 42:115–118. 10.1128/jcm.42.1.115-118.2004 14715740 10.1128/JCM.42.1.115-118.2004 PMC321727

[22] Almirante B, Rodríguez D, Cuenca-Estrella M, Almela M, Sanchez F, Ayats J et al (2006) Epidemiology, risk factors, and prognosis of Candida parapsilosis bloodstream infections: case-control population-based surveillance study of patients in Barcelona, Spain, from 2002 to 2003. J Clin Microbiol 44:1681–1685. 10.1128/jcm.44.5.1681-1685.2006 16672393 10.1128/JCM.44.5.1681-1685.2006 PMC1479182

[23] Held J, Wagner D (2011) β-d-glucan kinetics for the assessment of treatment response in Pneumocystis jirovecii pneumonia. Clin Microbiol Infect 17:1118–1122. 10.1111/j.1469-0691.2010.03452.x 21446990 10.1111/j.1469-0691.2010.03452.x

[24] Mori T, Ikemoto H, Matsumura M, Yoshida M, Inada K, Endo S et al (1997) Evaluation of plasma (1–>3)-beta-D-glucan measurement by the kinetic turbidimetric Limulus test, for the clinical diagnosis of mycotic infections. Eur J Clin Chem Clin Biochem 35:553–560 9263735

