# Supplementary material for: Evaluating the sensitivity of the Wako β-D-glucan assay for the diagnosis of candidemia caused by Candida parapsilosis
Source: Eur J Clin Microbiol Infect Dis. 2026 Mar 4;45(6):1683–92. doi: 10.1007/s10096-026-05446-z (PMC13319990; doi:10.1007/s10096-026-05446-z)
Supplement: Supplementary file 1 — Supplementary Material 1 (DOCX 18.9 KB) [file 10096_2026_5446_MOESM1_ESM.docx]

**Online Resource 1.** Distribution of *Candida* species

| *Candida* species | n |  |
| --- | --- | --- |
| *C. albicans* | 77 | 50% |
| *C. glabrata* | 24 | 16% |
| *C. parapsilosis* | 24 | 16% |
| *C. tropicalis* | 12 | 7.8% |
| *C. krusei* | 6 | 3.9% |
| *C. guilliermondii* | 5 | 3.2% |
| *C. dubliniensis* | 2 | 1.3% |
| Other *Candida* spp. | 4 | 2.6% |
| Total | 154 | 100% |

**Online Resource 2.** Summary of multiple candidemia episodes per patient

| Number of candidemia episodes per patient | Number of patients (n) | *Candida* species involved | Number of episodes |
| --- | --- | --- | --- |
| 6 times | 1 | *C. albicans* | 2 |
|  |  | *C. guilliermondii* | 2 |
|  |  | *C. parapsilosis* | 1 |
|  |  | *C. pelliculosa* | 1 |
| 3 times | 1 | *C. albicans* | 2 |
|  |  | *C. parapsilosis* | 1 |
| 2 times | 7 |  |  |
| episodes involving the same species | 6 | *C. albicans* | 6 |
|  |  | *C. glabrata* | 2 |
|  |  | *C. parapsilosis* | 2 |
|  |  | *C. tropicalis* | 2 |
| episodes involving different species | 1 | *C. albicans* | 1 |
|  |  | *C. glabrata* | 1 |
| Total | 9 |  | 23 |
